# Histone post-translational modifications in diabetic foot ulcer healing: linking immune dysregulation to tissue repair

**DOI:** 10.3389/fimmu.2026.1957976

**Published:** 2026-09-30

**Authors:** JingBo Hou, JunZhe Chen, YueZhong Chen, ZiYi Luo, Shaoxiang Yuan, Chengliang Deng, Shune Xiao

**Affiliations:** Department of Burns and Plastic Surgery, Affiliated Hospital of Zunyi Medical University, Zunyi, Guizhou, China

**Keywords:** angiogenesis, diabetic foot ulcer, epigenetic regulation, histone post-translational modifications, inflammation resolution, macrophages, therapeutic delivery, wound healing

## Abstract

Diabetic foot ulcer healing is compromised because metabolic, inflammatory and tissue-repair processes fail to proceed in a coordinated and timely manner. Persistent hyperglycemia, metabolic stress and oxidative imbalance can reshape immune-cell function and the tissue microenvironment, thereby sustaining inflammatory activation and hindering the transition to a reparative state. However, the epigenetic mechanisms that maintain these maladaptive states and link them to defective tissue regeneration remain incompletely understood. Histone post-translational modifications (hPTMs) constitute a dynamic regulatory layer through which metabolic and inflammatory signals are integrated with chromatin structure and gene expression. Persistent alterations in hPTMs may contribute to molecular imprints associated with metabolic memory, thereby sustaining vascular inflammation and remodeling that may further compromise tissue repair in diabetic foot ulcers. This Review summarizes current evidence for the involvement of histone acetylation, methylation, phosphorylation and metabolism-associated histone modifications in immune dysregulation and impaired diabetic foot ulcer healing. We discuss how hPTM-dependent mechanisms regulate inflammatory activation and resolution, macrophage functional transitions and vascular remodeling, and how these alterations influence angiogenesis, fibroblast-to-myofibroblast differentiation, extracellular matrix remodeling and keratinocyte-mediated re-epithelialization. Particular attention is given to the distinction between evidence derived directly from diabetic foot ulcer studies and evidence extrapolated from other diabetic tissues, non-diabetic wound models and *in vitro* systems. We also discuss recent direct diabetic wound-model interventions targeting acetylation-related pathways, including the p300/CBP–ZEB2 axis and restoration of H3K27ac-associated macrophage regulatory states, as well as lactate-oxidizing strategies that modulate lactylation-related responses. These studies provide intervention-based preclinical support for the functional involvement of acetylation and metabolism-associated chromatin regulation in diabetic wound repair, although the responsible histone sites, causal cell populations, treatment windows and relevance to human DFUs remain incompletely defined. Taken together, current evidence supports hPTMs as an important regulatory interface linking the diabetic microenvironment to persistent immune dysregulation, vascular dysfunction and impaired tissue repair. However, the therapeutic relevance of these mechanisms remains difficult to assess because the available evidence is heterogeneous and predominantly preclinical, while the cell specificity, temporal dynamics and clinical applicability of epigenetic interventions remain uncertain.

## 1 Introduction

Diabetes mellitus is a metabolic disorder characterized primarily by chronic hyperglycemia and is frequently accompanied by multisystem abnormalities, including insulin resistance, immune dysregulation and vascular injury (1). As of 2025, diabetes continues to impose a substantial and growing global health burden. According to the International Diabetes Federation, approximately 589 million adults aged 20–79 years worldwide are living with diabetes, corresponding to a prevalence of approximately 11.1%. Diabetic foot ulceration (DFU) is one of the most serious chronic complications of diabetes, with an estimated global prevalence of approximately 6.3% among individuals with diabetes (2). Its pathogenesis involves multiple diabetes-related abnormalities, including chronic hyperglycemia, insulin resistance and dysregulation of the immune microenvironment (3). Individuals with DFU are at increased risk of infection, progressive tissue ulceration and, in severe cases, gangrene, all of which can substantially impair quality of life (4). The clinical recalcitrance of DFUs is largely attributable to impaired wound healing. Wound healing is a complex, highly coordinated and dynamic process comprising four partially overlapping phases—hemostasis, inflammation, proliferation and remodeling—that progress in a broadly sequential manner while remaining functionally interdependent (5, 6). During the inflammatory phase, for example, circulating monocytes are recruited to the wound and differentiate into macrophages that help eliminate pathogens, necrotic tissue and cellular debris. Subsequently, signals within the local microenvironment promote the gradual transition of macrophages from a pro-inflammatory state towards a reparative state, thereby facilitating the transition from inflammation to tissue formation (7). In diabetes, persistent inflammation, metabolic disturbances and vascular dysfunction disrupt this coordinated repair program and ultimately contribute to impaired wound healing.

However, inflammatory, vascular and cellular abnormalities alone do not fully account for the persistent aberrant transcriptional programs observed in chronic diabetic wounds. Consequently, increasing attention has focused on histone post-translational modifications (hPTMs) as potential contributors to this pathological state. As important epigenetic regulators of chromatin organization and gene transcription, hPTMs can influence the expression of genes encoding inflammatory cytokines, angiogenic factors, extracellular matrix components and regulators of cell migration by altering nucleosome conformation, modulating chromatin accessibility and recruiting specific effector proteins (8, 9).

Against this background, this review examines the mechanisms through which hPTMs may contribute to impaired healing in diabetic foot ulcers. Particular emphasis is placed on their effects on inflammation resolution and macrophage functional states, angiogenesis, fibroblast and myofibroblast function, extracellular matrix remodeling and re-epithelialization. In addition, this review distinguishes evidence derived directly from DFU studies from indirect evidence obtained from other diabetic tissues, *in vitro* cell systems and non-DFU animal models. By integrating these different levels of evidence, we aim to clarify the current mechanistic understanding of hPTM dysregulation in diabetic wound healing and to identify priorities for future research and the development of targeted therapeutic strategies.

Compared with previous reviews, the present review adopts a multicellular and translational framework to examine how hPTMs may link immune dysregulation to defective tissue repair in DFUs. Rather than focusing predominantly on macrophage inflammation, we analyze hPTM-related mechanisms across the principal wound-healing compartments, including macrophages and neutrophils, endothelial cells, fibroblasts and myofibroblasts, keratinocytes and the extracellular matrix. These cellular compartments are considered in relation to the partially overlapping phases of hemostasis, inflammation, proliferation and remodeling.

A further objective of this review is to distinguish the biological plausibility of a mechanism from the directness and translational maturity of the supporting evidence. By integrating cellular compartments, wound-healing stages and evidence directness, this framework identifies where hPTM mechanisms are supported by intervention studies and where they remain associative, indirect or hypothesis-driven. It is intended to prevent findings from other tissues or experimental systems from being interpreted as established therapeutic mechanisms in human DFUs.

For the purposes of this review, evidence was classified according to biological directness, evidence type and translational maturity. Direct human DFU-associated evidence refers to studies using human DFU tissue, wound fluid, circulating cells or other clinical samples to evaluate hPTM-related changes or associations. Such studies provide direct disease-context evidence but may be observational and do not necessarily establish causality or therapeutic efficacy. Direct diabetic wound-model evidence refers to mechanistic or intervention-based studies conducted in diabetic animal wound models. These studies may provide causal preclinical evidence but do not constitute clinical evidence in patients with DFUs. Indirect wound-repair or mechanistic evidence refers to studies conducted in non-diabetic cutaneous wounds, other diabetic tissues, ex vivo systems or isolated wound-relevant cells. External clinical evidence refers to clinical studies in unrelated diseases that provide information regarding systemic exposure, safety, local delivery or translational feasibility, but not DFU efficacy. Within each category, intervention-based, mechanistic, associative and hypothesis-driven evidence were distinguished where applicable. Translational maturity was considered according to the progression from molecular or cellular observations to intervention-based animal studies, locally delivered therapeutic platforms, human ex vivo models and clinical studies. This classification was applied consistently throughout the main text, **Figure 1** and **Table 1**.

**Figure 1:**
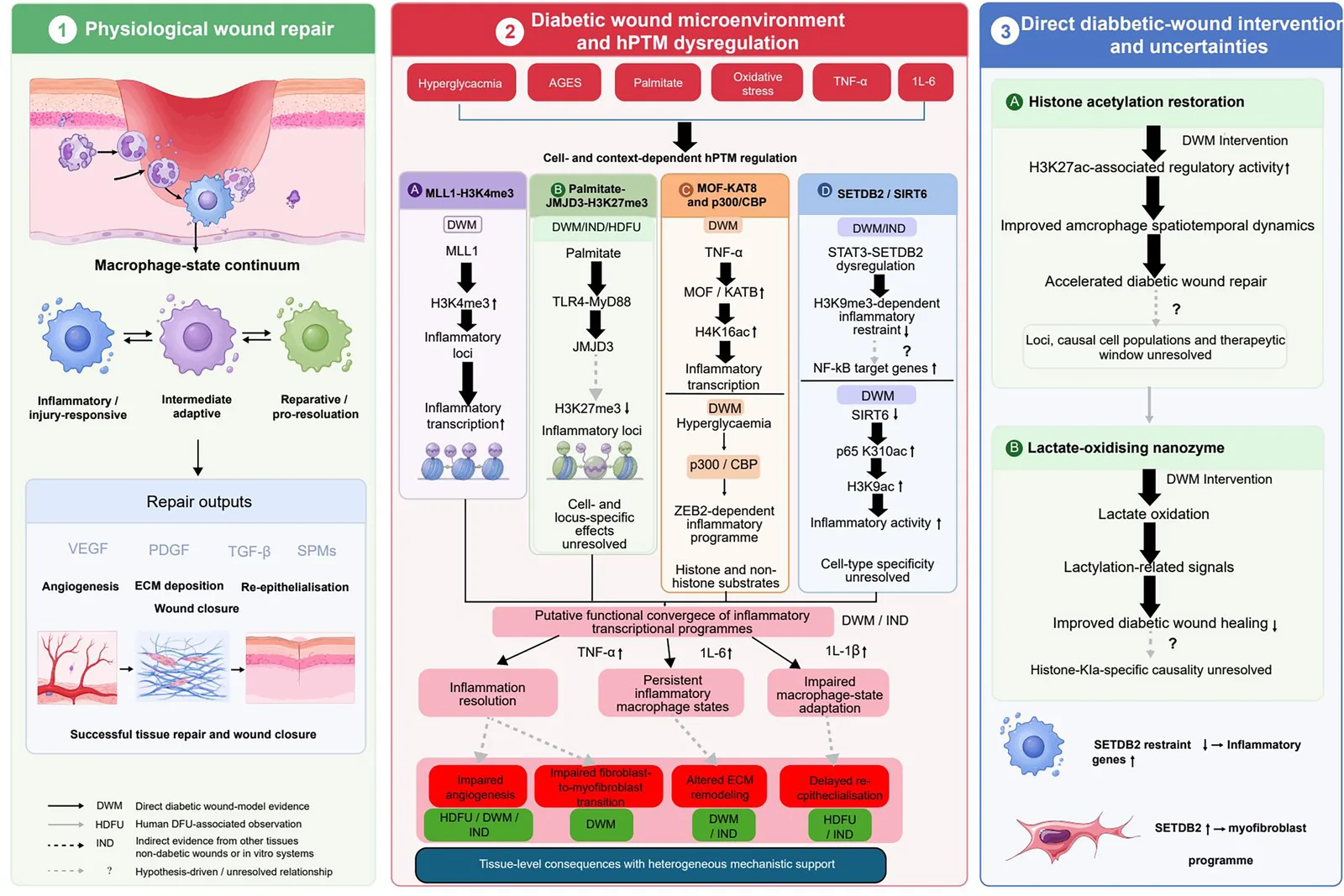
Conceptual framework linking diabetes-associated hPTM dysregulation to impaired immune resolution and tissue repair. The pathways shown are selected representative mechanisms and are not intended to provide an exhaustive summary of all histone post-translational modification (hPTM)-related pathways discussed in the manuscript. Under physiological conditions, apoptotic neutrophil clearance and local tissue-derived signals support dynamic changes across a macrophage-state continuum, including inflammatory or injury-responsive, intermediate or adaptive, and reparative or pro-resolution states. These states are functional descriptors rather than discrete or terminal M1/M2 lineages. Their relative abundance and activities may vary with wound-healing stage, anatomical location and microenvironment. In diabetic wounds, hyperglycemia, advanced glycation end products (AGEs), palmitate, oxidative stress and inflammatory mediators may disturb macrophage signaling and hPTM regulation. Representative mechanisms include MLL1-associated H3K4me3 enrichment, palmitate–TLR4/MyD88–JMJD3 signaling with reduced H3K27me3, TNF-alpha–MOF/KAT8-associated H4K16ac, dysregulated STAT3–SETDB2 signaling, reduced SIRT6-associated deacetylation of H3K9ac and NF-kappaB p65 Lys310, and p300/CBP–ZEB2-associated inflammatory transcription. These alterations may contribute to persistent inflammatory transcription, reduced inflammation resolution and impaired adaptation across macrophage functional states. The resulting changes in inflammatory and pro-repair signaling may be associated with defective angiogenesis, fibroblast-to-myofibroblast transition, ECM remodeling, re-epithelialization and diabetic wound repair. The figure also shows selected direct diabetic-wound interventions, including restoration of histone acetylation and a lactate-oxidizing nanozyme. These interventions improved wound repair in preclinical diabetic wound models, but their precise causal cell populations, target loci, temporal windows and clinical relevance remain unresolved. In particular, reduced lactylation-related signals following lactate oxidation do not by themselves establish histone-Kla-dependent causality. The opposing effects of SETDB2 in macrophages and fibroblasts further illustrate the cell-type dependence of hPTM regulation. The integrated model combines evidence from human DFU-associated observations, diabetic wound models, other diabetic tissues, non-diabetic wound models and *in vitro* systems. Solid arrows indicate direct or experimentally supported relationships, dashed arrows indicate indirect, associative or cross-model relationships, and dotted arrows with question marks indicate unresolved or hypothesis-driven relationships. The model is not intended to imply that all pathways operate simultaneously or have been fully validated in primary human DFU tissue. AGEs, advanced glycation end products; CBP, CREB-binding protein; DWM, direct diabetic wound-model evidence; ECM, extracellular matrix; DFU, diabetic foot ulcer; H3K4me3, histone H3 lysine 4 trimethylation; H3K9ac, histone H3 lysine 9 acetylation; H3K9me3, histone H3 lysine 9 trimethylation; H3K27ac, histone H3 lysine 27 acetylation; H3K27me3, histone H3 lysine 27 trimethylation; H4K16ac, histone H4 lysine 16 acetylation; HDFU, human diabetic foot ulcer-associated evidence; hPTM, histone post-translational modification; IL, interleukin; IND, indirect evidence; JAK, Janus kinase; KAT8, lysine acetyltransferase 8; Kla, lysine lactylation; MLL1, mixed-lineage leukemia 1; MOF, males absent on the first; MyD88, myeloid differentiation primary response 88; NF-kappaB, nuclear factor kappa B; PDGF, platelet-derived growth factor; SIRT6, sirtuin 6; SPMs, specialized pro-resolving mediators; STAT3, signal transducer and activator of transcription 3; SETDB2, SET domain bifurcated histone lysine methyltransferase 2; TGF-beta, transforming growth factor-beta; TLR4, Toll-like receptor 4; TNF-alpha, tumor necrosis factor-alpha; VEGF, vascular endothelial growth factor; ZEB2, zinc finger E-box binding homeobox 2.

**Table 1:** Histone modification-, metabolism- and epigenetic platform-related evidence and interventions in diabetic wound repair.

Evidence category, evidence type and representative strategy	Principal target or mode of action	Research model and tissue context	Main findings	Translational interpretation and limitations	References
Direct human DFU-associated evidence					
HDAC- and sirtuin-related expression profiling; associative observational evidence	Association of HDAC and sirtuin expression with NRF2-mediated redox regulation and angiogenic signalling	Wound tissues from patients with DFUs of different clinical grades	HDAC1, HDAC3, HDAC4, HDAC11 and several sirtuins showed grade-dependent expression changes. Increased HDAC4 expression was associated with reduced NRF2 and angiogenesis-related markers, including VEGF, HIF-1alpha and SDF-1alpha.	Provides direct human DFU disease-context evidence but does not establish causality, histone-site-specific mechanisms or therapeutic efficacy. The cellular origin of the signals and their longitudinal relationship with healing remain unclear.	(94, 102)
Direct diabetic wound-model evidence					
Macrophage-specific JMJD3 inhibition; intervention-based and mechanistic evidence	Inhibition of JMJD3-associated demethylation and inflammatory transcriptional programmes	Diabetic mouse wound models; macrophage-targeted genetic, pharmacological or nanoparticle-based intervention	JMJD3 inhibition reduced inflammatory signalling, including STING-associated responses, and was associated with improved diabetic wound healing.	Supports a macrophage-directed preclinical strategy. The relative contribution of JMJD3 inhibition in macrophages and other wound-resident cells, target engagement, optimal treatment window and relevance to human DFUs remain uncertain.	(91, 92)
DZ2002; pharmacological intervention-based evidence	Modulation of the MLL1-H3K4me3 axis through inhibition of S-adenosylhomocysteine hydrolase	Diabetic animal wound models; macrophage-associated inflammatory responses	DZ2002 attenuated persistent macrophage inflammatory programmes and was associated with improved wound healing.	Supports pharmacological modulation of methylation-associated inflammation. The compound may affect pathways beyond a single chromatin target; pharmacokinetics, systemic effects, target specificity and long-term safety require evaluation.	(126)
C646; local intervention-based and mechanistic evidence	Inhibition of p300/CBP acetyltransferase activity and modulation of the p300/CBP-ZEB2 axis	Diabetic mouse wounds; local treatment at wound margins	Local C646 treatment attenuated ZEB2-associated inflammatory macrophage responses and was associated with accelerated wound closure.	Provides direct preclinical evidence for p300/CBP-related regulation in diabetic wounds. Because p300/CBP modifies multiple histone and non-histone substrates, the effect cannot be attributed exclusively to one histone acetylation site.	(97)
Restoration of histone acetylation with butyrate; metabolic-intervention-based evidence	Restoration of acetylation-associated macrophage regulatory activity, including H3K27ac-associated programmes	Diabetic wound models and related cellular systems	Butyrate-based treatment improved the spatiotemporal organisation of macrophage responses and was associated with improved coordination among macrophages, keratinocytes and fibroblasts during wound repair.	Represents an upstream metabolic-epigenetic intervention rather than a highly selective enzyme-targeted treatment. Contributions from H3K27ac, non-histone acetylation, metabolism and other butyrate-responsive pathways remain unresolved.	(98)
SIRT6-directed intervention; deacetylation-based mechanistic and pharmacological evidence	Modulation of the SIRT6-NF-kappaB p65 axis and associated histone acetylation	Diabetic wound models; macrophage-associated inflammatory responses	SIRT6 activation or pharmacological stabilisation reduced p65 Lys310 acetylation and H3K9ac-associated inflammatory activity and was associated with improved diabetic wound repair.	Provides preclinical support for SIRT6-associated deacetylation. Human DFU findings are mainly associative, and the relative importance of histone versus non-histone substrates, responsible cell populations and treatment timing remains to be established.	(94–96)
Lactate-oxidising single-atom nanozyme (Fe@CN-P); metabolic-intervention-based evidence	Promotion of lactate oxidation and metabolic reprogramming, with associated reduction in lactylation-related signals	Diabetic wound animal models; metabolically stressed wound microenvironment	The nanozyme promoted lactate oxidation, altered the wound metabolic microenvironment, reduced lactylation-related signals and improved wound healing.	Provides direct preclinical evidence that lactate metabolism can influence lactylation-associated responses. It does not establish that histone lactylation is the necessary or direct mediator of the healing effect. Effects on oxidative metabolism, reactive oxygen species and other pathways must also be considered.	(99)
Silk-fibroin core-shell microneedle patch containing a BRD9 degrader and Hedgehog agonist; local sequential intervention-based evidence	Sequential modulation of chromatin-associated BRD9 activity and Hedgehog signalling	Diabetic wound animal models; locally delivered microneedle platform	Sequential release attenuated excessive early inflammation and subsequently promoted re-epithelialisation, collagen deposition and angiogenesis.	Demonstrates the potential value of stage-specific local delivery. The individual contributions of BRD9 degradation and Hedgehog activation, tissue penetration, release reproducibility, manufacturing consistency and safety require further study.	(127)
Sprayable epigenetically active nanozyme hydrogel; local intervention-based evidence	Local remodelling of inflammatory chromatin states and modulation of macrophage responses under oxidative stress	Diabetic wound-related cellular and animal models	The hydrogel produced sustained anti-inflammatory and pro-regenerative effects and was associated with reduced inflammatory macrophage programmes and improved wound repair.	Supports the feasibility of local and sustained delivery. Cell specificity, molecular target engagement, release kinetics, biodistribution, degradation products and local and systemic safety remain to be established.	(128)
Indirect wound-repair, tissue or mechanistic evidence					
HDAC and sirtuin modulation; indirect intervention-based evidence	Modulation of histone acetylation and deacetylation during cutaneous repair	Non-diabetic skin-repair models	Histone acetylation and sirtuin activity were associated with changes in skin repair, indicating that HDAC- and sirtuin-related pathways may exert context-dependent effects on tissue regeneration.	Provides indirect wound-repair evidence only. The direction of effect may depend on the specific inhibitor, cell type and healing stage; these findings do not establish a DFU-specific therapeutic strategy.	(130, 131)
TSA; indirect intervention-based and mechanistic evidence	Broad HDAC inhibition and modulation of histone acetylation and myeloid-cell plasticity	Acute cutaneous injury and related cellular or tissue-regeneration models	TSA was associated with enhanced epidermal and dermal regeneration, increased keratinocyte proliferation, expansion of stem/progenitor-cell populations and altered myeloid-cell behaviour.	Provides indirect evidence that broad HDAC modulation can influence tissue repair. Non-selective effects on histone and non-histone substrates and stage- and cell-type-dependent responses limit extrapolation to DFU therapy.	(124, 125)
Exosomes from TSA-pretreated bone-marrow mesenchymal stem cells; indirect combined intervention	Epigenetic cell pretreatment combined with exosome-mediated paracrine delivery	Cellular experiments and non-diabetic animal wound models	The exosomes were associated with reduced inflammatory macrophage programmes, enhanced endothelial-cell migration and angiogenesis, increased collagen-associated repair responses and faster wound healing.	Illustrates a combined cell-preconditioning and extracellular-vesicle strategy. The independent contributions of TSA pretreatment, exosomal cargo and host-cell responses are difficult to separate, and efficacy in human DFUs has not been established.	(132)
Sodium 4-phenylbutyrate; indirect ex vivo intervention	Modulation of endoplasmic-reticulum stress, with possible secondary effects on HDAC-related processes	Keratinocytes from older donors and an ex vivo human skin-wound model	4-Phenylbutyrate restored keratinocyte migratory capacity and promoted re-epithelialisation.	Provides human-cell and ex vivo skin evidence for a possible epithelial-repair effect. The benefit cannot be attributed specifically to HDAC inhibition, and efficacy in diabetic, ischemic or infected foot wounds remains untested.	(133)
Indirect evidence from other tissues or non-wound models					
Sodium valproate; indirect mechanistic evidence	Broad modulation of HDAC activity and histone acetylation	Amphibian tail-regeneration model and mouse cell experiments	Valproate produced divergent effects across models, including inhibition of amphibian tail regeneration and reduced apoptosis in mouse cells.	Illustrates the tissue-, species- and context-dependence of HDAC-directed interventions. These findings should not be interpreted as evidence of efficacy in diabetic wounds.	(124)
HDAC inhibition in postoperative ocular scarring; indirect intervention-based evidence	Modulation of histone acetylation and fibrotic responses	Rabbit model of postoperative ocular scarring following glaucoma filtration surgery	HDAC inhibition reduced collagen deposition, F-actin and alpha-SMA expression and excessive postoperative scar formation.	Provides indirect evidence for an antifibrotic effect in a postoperative ocular model. The tissue, disease mechanism and therapeutic endpoint differ substantially from those of chronic DFUs.	(134)
External clinical evidence for safety, delivery or feasibility, not DFU efficacy					
Tazemetostat; external clinical safety and systemic-exposure evidence	Systemic inhibition of EZH2	First-in-human phase I oncology study	Tazemetostat showed clinical activity in selected malignancies but was associated with treatment-related adverse events and dose-related toxicity.	Provides indirect information regarding systemic exposure, tolerability and the risks of pharmacological epigenetic modulation. It does not provide evidence of therapeutic efficacy in DFUs.	(135)
Remetinostat; external local-delivery and safety evidence	Topical HDAC inhibition	Phase II study in patients with basal cell carcinoma	Topical remetinostat showed local clinical activity without observed systemic adverse events in the reported exploratory study.	Supports the feasibility of local epigenetic delivery and the potential to limit systemic exposure. Tumour biology and treatment endpoints differ from those of diabetic wounds, and no DFU efficacy can be inferred.	(136)
SB939; external clinical safety and feasibility evidence	Systemic pan-HDAC inhibition	Phase II oncology study in translocation-associated recurrent or metastatic sarcomas	SB939 produced disease stabilisation in some patients but did not demonstrate confirmed objective responses.	Provides limited information regarding systemic tolerability and clinical development of pan-HDAC inhibition. It does not establish efficacy in DFUs and reinforces the need for disease-specific target selection and delivery strategies.	(137)

α-SMA, α-smooth muscle actin; BRD9, bromodomain-containing protein 9; CBP, CREB-binding protein; DFU, diabetic foot ulcer; HDAC, histone deacetylase; H3K4me3, histone H3 lysine 4 trimethylation; H3K27ac, histone H3 lysine 27 acetylation; JMJD3, Jumonji domain-containing protein 3; MLL1, mixed-lineage leukemia 1; p300, histone acetyltransferase p300; STING, stimulator of interferon genes; TSA, trichostatin A; ZEB2, zinc-finger E-box-binding homeobox 2.

Evidence categories reflect biological proximity to diabetic foot-ulcer (DFU) pathology, evidence type and translational context. “Direct human DFU-associated evidence” refers to studies using human DFU tissue, wound fluid, circulating cells or other clinical samples to evaluate hPTM-related changes or associations. Such studies provide direct disease-context evidence but may be observational and do not necessarily establish causality or therapeutic efficacy. “Direct diabetic wound-model evidence” refers to mechanistic or intervention-based studies conducted in diabetic animal wound models. These studies may provide causal preclinical evidence but do not constitute clinical evidence in patients with DFUs. “Indirect wound-repair, tissue or mechanistic evidence” refers to studies conducted in non-diabetic cutaneous wounds, other diabetic tissues, ex vivo systems or isolated cells. “External clinical evidence” refers to clinical studies in unrelated diseases that provide information about systemic exposure, safety, local delivery or translational feasibility, but not DFU efficacy. Within each category, intervention-based, mechanistic, associative and hypothesis-driven evidence were distinguished where applicable. No hPTM-targeted intervention listed in this table has established clinical efficacy in patients with DFUs. The effects of the listed interventions may involve histone and non-histone substrates, multiple signaling pathways or metabolic changes and should therefore be interpreted according to the model, cell type, treatment timing and endpoint examined.

### 1.1 Literature search and evidence classification

To identify relevant literature, we searched the PubMed database from database inception through 25 August 2026. The search strategy combined terms related to diabetic foot ulcers and diabetic wounds, including “diabetic foot ulcer*”, “diabetic wound*”, “diabetes” and “wound healing”, with terms related to histone post-translational modifications and chromatin regulation, including “histone modification*”, “histone acetylation”, “histone methylation”, “histone phosphorylation”, “histone lactylation”, “histone citrullination”, “NETosis”, “histone deacetylase”, “histone acetyltransferase”, “histone methyltransferase”, “histone demethylase”, “sirtuin”, “SETDB2”, “MLL1”, “JMJD3”, “MOF”, “p300/CBP” and “chromatin”. Additional relevant publications were identified by manually screening the reference lists of retrieved primary studies and relevant reviews.

We prioritized original studies that investigated histone modifications, histone-modifying enzymes or chromatin-associated mechanisms in diabetic wounds, human DFU tissue, diabetic wound models or wound-relevant cell types. Studies conducted in non-diabetic cutaneous wounds, other diabetic tissues, isolated cells or ex vivo systems were included when they provided relevant mechanistic, therapeutic, safety or drug-delivery information. Clinical studies in unrelated diseases were considered only when they provided contextual information regarding the safety, systemic exposure, route of administration or translational feasibility of epigenetic interventions. General studies of chromatin biology were used to explain the molecular functions of hPTMs but were not considered direct evidence of DFU pathology. Studies were selected when they were relevant to the review objectives and provided identifiable information regarding the investigated hPTM or chromatin regulator, experimental model, cell or tissue compartment, wound-healing outcome or translational implication. Publications without a clear connection to wound repair, diabetic complications or the interpretation of hPTM-based therapeutic strategies were generally not prioritized, except when they were required to explain fundamental chromatin mechanisms. This search and selection process was intended to support a narrative, evidence-informed review rather than a systematic review or meta-analysis.

## 2 Histone post-translational modifications in physiological wound healing

### 2.1 Overview of wound healing

Normal wound repair depends on the coordinated progression of hemostasis, inflammation, tissue formation and extracellular matrix (ECM) remodeling. Although these processes are temporally distinguishable, they partially overlap and remain functionally interdependent. Their progression is regulated by dynamic interactions among multiple cell types, soluble mediators and matrix components (**Figure 2**).

**Figure 2:**
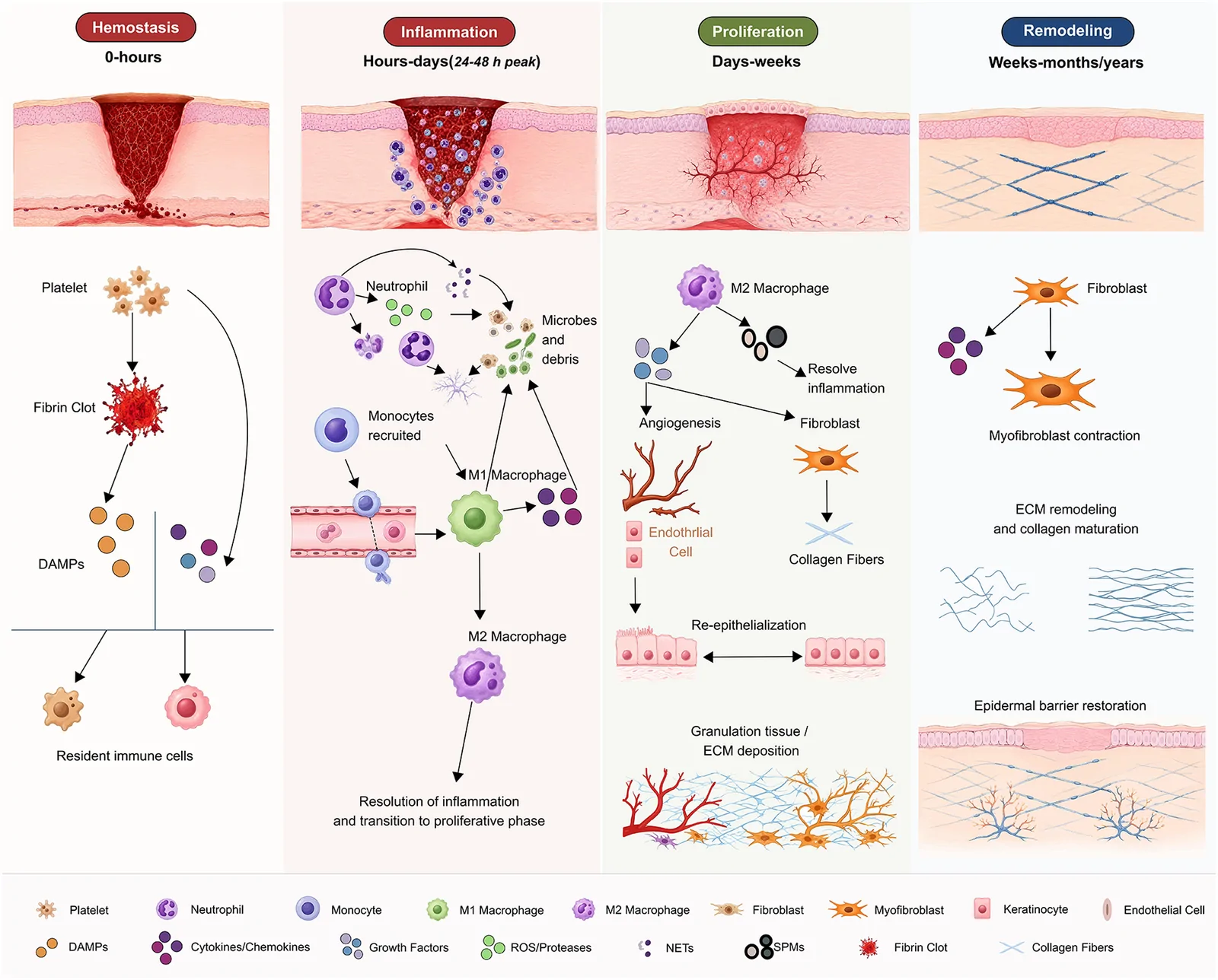
Stages of normal cutaneous wound healing and the major cellular events. Wound healing proceeds through four stages: hemostasis, inflammation, proliferation, and remodeling, which are sequentially connected and partially overlap in time. During hemostasis, platelets aggregate and form a fibrin clot, while damage-associated molecular patterns (DAMPs) initiate the local inflammatory response. During inflammation, neutrophils and monocytes are recruited to the wound, and macrophages acquire temporally and spatially distinct inflammatory, reparative and intermediate functional states that contribute to inflammation resolution and tissue repair. The proliferative phase is characterized primarily by angiogenesis, fibroblast activation, extracellular matrix (ECM) deposition, and re-epithelialization. The remodeling phase involves myofibroblast contraction, collagen fiber reorganization, and ECM maturation. Arrows indicate the relationships among the major cellular and tissue repair events. ROS, reactive oxygen species; NETs, neutrophil extracellular traps; SPMs, specialized pro-resolving mediators; VEGF, vascular endothelial growth factor; TGF-β, transforming growth factor-β; PDGF, platelet-derived growth factor; MMPs, matrix metalloproteinases; α-SMA, α-smooth muscle actin.

#### 2.1.1 Hemostasis and initiation of inflammation

Immediately after tissue injury, platelet adhesion and aggregation initiate the coagulation cascade, leading to the formation of a fibrin-rich clot. In addition to limiting blood loss, the clot provides a temporary scaffold for the recruitment and migration of reparative cells (10–12). Damaged cells simultaneously release damage-associated molecular patterns (DAMPs), which act together with platelet-derived chemokines, growth factors and inflammatory mediators to initiate local immune activation (7, 9, 11, 12). Neutrophils are among the earliest immune cells recruited to the wound. They contribute to early host defense by eliminating pathogens and damaged tissue and by releasing reactive oxygen species (ROS), proteases and inflammatory mediators. Neutrophils may also form neutrophil extracellular traps (NETs), which help restrict the spread of invading microorganisms (7, 13–16). As the inflammatory response develops, circulating monocytes are recruited through chemokine pathways, including the C-C motif chemokine ligand 2 (CCL2)–C-C motif chemokine receptor 2 (CCR2) axis, and subsequently differentiate into macrophages (17, 18). At this stage, macrophages commonly display a pro-inflammatory functional state and contribute to pathogen elimination, tissue-debris clearance and the amplification of local inflammation (7).

#### 2.1.2 Resolution phase of inflammation

The transition from inflammation to tissue repair requires the timely termination of the initial innate immune response. After completing their early defense functions, neutrophils undergo apoptosis and are removed by macrophages through efferocytosis. This process, together with signals from the local tissue microenvironment, promotes a shift in macrophage function from a pro-inflammatory state towards a reparative state (7, 12, 19). Reparative macrophages release vascular endothelial growth factor (VEGF), transforming growth factor-β (TGF-β), platelet-derived growth factor (PDGF) and other mediators that support angiogenesis, fibroblast activation and ECM deposition. These activities help establish the tissue conditions required for the subsequent proliferative phase (17, 19, 20). In parallel, specialized pro-resolving mediators (SPMs), including lipoxins, resolvins, protectins and maresins, actively contribute to the termination of inflammation and the restoration of a tissue microenvironment that favors repair (21, 22).

#### 2.1.3 Proliferative phase

Once the inflammatory response has been sufficiently controlled, the wound enters a phase dominated by tissue formation and closure. Endothelial cells, keratinocytes and fibroblasts become activated and coordinate angiogenesis, re-epithelialization, granulation-tissue formation and ECM remodeling (23–25). Fibroblasts produce collagen, fibronectin and other matrix components that provide structural support for newly formed tissue. At the same time, matrix metalloproteinases (MMPs) and other proteases mediate the degradation and replacement of the provisional matrix. The balance between matrix deposition and degradation allows granulation tissue to mature and contributes to progressive wound closure (7, 18, 26).

#### 2.1.4 Remodeling phase

Remodeling generally begins after the establishment of granulation tissue and may continue for several months or even years (12). In response to TGF-β, mechanical tension and other local cues, a subset of fibroblasts differentiates into α-smooth muscle actin (α-SMA)-positive myofibroblasts. Through their contractile activity, these cells contribute to wound contraction and closure (27). During this phase, the ECM deposited during the early stages of repair is continuously degraded, reorganized and replaced. Type III collagen is gradually reduced and replaced by a greater proportion of type I collagen, resulting in a more mature matrix architecture and improved mechanical properties of the repaired tissue. The quality of remodeling depends on the balance among collagen turnover, matrix reorganization and the timely resolution of myofibroblast activity.

### 2.2 Types of hPTMs

Three principal classes of regulatory factors — writers, readers and erasers — coordinate the deposition, recognition and removal of histone post-translational modifications (hPTMs) (8, 28). Writers catalyse the addition of specific histone modifications at defined residues. For example, EP300/CBP catalyzes histone acetylation, thereby increasing chromatin accessibility and facilitating gene transcription, whereas SETD2 is the principal methyltransferase responsible for H3K36 trimethylation. In addition to catalyzing H3K36me3 deposition, SETD2 participates in transcriptional elongation, RNA processing and DNA damage repair (29). Readers recognize histone modification marks through specific structural domains and recruit downstream effector complexes, thereby translating epigenetic signals into transcriptional regulatory outputs (8, 28). Erasers remove pre-existing histone modifications, thereby maintaining hPTM states in a dynamic and reversible manner. For example, HDAC3-mediated removal of histone acetyl groups is generally associated with chromatin compaction and transcriptional repression (30). By contrast, KDM5B, a histone lysine demethylase, primarily removes active methylation marks, including H3K4me3 and H3K4me2, thereby contributing to gene repression (31). Collectively, writers, readers and erasers form a dynamic regulatory network through which histone modifications influence transcriptional programs and cellular functional states (8, 28).

#### 2.2.1 Histone acetylation

Histone acetylation occurs predominantly at lysine residues within the N-terminal tails of histones H3 and H4 and is among the most extensively studied hPTMs. Advances in mass spectrometry and other proteomic technologies have identified an increasing number of histone lysine acetylation sites, highlighting the complexity and site specificity of this modification. Histone acetylation generally neutralizes the positive charge of lysine residues, thereby weakening histone-DNA interactions, promoting a more open chromatin conformation and facilitating recruitment of transcription-associated protein complexes (8, 32).

Histone acetyltransferases (HATs) and histone deacetylases (HDACs) are the principal enzymes that dynamically regulate histone acetylation (33, 34). HATs catalyze the transfer of acetyl groups from acetyl coenzyme A (acetyl-CoA) to the ϵ-amino groups of histone lysine residues, thereby promoting histone acetylation (33). Acetyl-CoA is a central metabolite that links glucose, fatty acid and amino acid metabolism. Accordingly, changes in acetyl-CoA availability can couple cellular metabolic state to chromatin modification and gene regulation (35). Histone acetylation-related enzymes and reader proteins identified to date are summarized in **Table 2**

**Table 2:** Histone acetylation-related enzymes and reader proteins.

Regulatory category	Representative members/domains	Key functional features	References
Writer (acetyltransferases)	KAT3A/CBP and KAT3B/p300	Transcriptional co-regulators that acetylate histone and non-histone substrates and can increase chromatin accessibility and transcriptional activity.	(33, 36)
	Mammalian MYST/KAT family members, including KAT5/TIP60, KAT6A/MOZ, KAT6B/MORF and KAT8/MOF	Catalyse site-specific histone acetylation and participate in chromatin regulation, DNA-damage responses and transcriptional control.	(33, 36)
	GNAT/KAT family: KAT1/HAT1, KAT2A/GCN5 and KAT2B/PCAF	Catalyse histone acetylation and regulate transcriptional programmes through chromatin-associated and, in some contexts, non-histone substrates.	(33, 36)
Eraser: histone deacetylases	HDAC1, HDAC2, HDAC3 and HDAC8 (class I)	Remove acetyl groups from histone and non-histone substrates. Their effects on chromatin accessibility and transcription depend on substrate selection, interacting complexes and cellular context.	(30, 34)
	HDAC4, HDAC5, HDAC7 and HDAC9 (class IIa)	Regulate transcription through interactions with transcription factors and co-repressor complexes; their functions are context dependent and include regulation of histone and non-histone acetylation-associated processes.	(30, 34)
	HDAC6/10 (class IIb)	Participate predominantly in the deacetylation of non-histone substrates and in the regulation of cellular stress and cytoplasmic processes.	(30, 34)
	SIRT1–SIRT7 (class III)	NAD+-dependent deacetylases and deacylases that link cellular metabolism to chromatin regulation, stress responses and transcriptional control.	(30, 34)
	HDAC11 (class IV)	A distinct histone deacetylase with context-dependent functions in transcriptional regulation and immune signalling.	(30, 34)
Reader (reader proteins)	Bromodomain-containing proteins	Recognise acetylated lysine residues and recruit transcriptional regulatory complexes, thereby linking histone acetylation to downstream chromatin and transcriptional responses.	(36, 37)

CBP, CREB-binding protein; GNAT, GCN5-related N-acetyltransferase; HAT1, histone acetyltransferase 1; HBO1, histone acetyltransferase binding to ORC1; HDAC, histone deacetylase; KAT, lysine acetyltransferase; MORF, monocytic leukemia zinc-finger-related factor; MOF, males absent on the first; MYST, MYST histone acetyltransferase family; PCAF, p300/CBP-associated factor; SIRT, sirtuin; TIP60, Tat-interactive protein 60 kDa.

The table summarizes representative histone acetylation-related enzymes and reader proteins based on established chromatin biology; inclusion in the table does not imply direct evidence of involvement in diabetic foot ulcer pathology.

#### 2.2.2 Histone methylation

Both arginine and lysine residues within histones can undergo methylation. In contrast to histone acetylation, which is generally associated with chromatin opening and transcriptional activation, the gene-regulatory effects of histone methylation depend on the modified residue, the specific site and the degree of methylation. Accordingly, histone methylation can either promote transcription or mediate transcriptional silencing. For example, H3K4me3 is generally associated with transcriptional activation, whereas H3K9me3 and H3K27me3 are more commonly associated with heterochromatin formation and gene silencing (8, 38). Methyltransferases and demethylases jointly regulate histone methylation states. Histone methylation-related enzymes and reader proteins identified to date are summarized in **Table 3**.

**Table 3:** Histone methylation-related enzymes and reader proteins.

Functional category	Representative enzymes/proteins	Key functional features	References
Writer: lysine methyltransferases	SUV39H1, SUV39H2, EZH2 and SETD2	Use S-adenosylmethionine as a methyl donor to methylate lysine residues. SUV39H enzymes catalyse H3K9 methylation; EZH2 catalyses H3K27 trimethylation within polycomb repressive complex 2; and SETD2 catalyses H3K36 trimethylation.	(29, 39, 40)
	DOT1L/KMT4	A non-SET-domain lysine methyltransferase that catalyses methylation of H3K79.	(41)
Writer: arginine methyltransferases	PRMT1, PRMT4/CARM1, PRMT5 and PRMT7	Use S-adenosylmethionine to catalyse histone and non-histone arginine monomethylation, asymmetric dimethylation or symmetric dimethylation. PRMTs are commonly classified as type I, II or III enzymes according to the methylarginine products generated.	(42)
Eraser: lysine demethylases	LSD1/KDM1A and LSD2/KDM1B	Flavin adenine dinucleotide-dependent amine oxidase-type demethylases that remove selected mono- and dimethylated lysine marks; KDM1A is best characterised in relation to H3K4me1/2 demethylation.	(43, 44)
	KDM2, KDM3, KDM4, KDM5 and KDM6 families	Fe2+- and 2-oxoglutarate-dependent Jumonji C-domain demethylases that remove specific lysine methylation marks; several family members can demethylate trimethylated substrates.	(43, 44)
Methylation-related enzyme with disputed demethylase activity	JMJD6	Initially reported to possess histone arginine demethylase activity; however, this classification remains controversial. Its more firmly supported activities include lysyl hydroxylation and functions linked to RNA-processing pathways.	(45, 46)
Methylation-antagonising or related enzyme	PADI4/PAD4	Catalyses arginine deimination (citrullination), which can antagonise histone arginine methylation but is mechanistically distinct from canonical demethylation. In neutrophils, PAD4-mediated histone citrullination contributes to chromatin decondensation during NET formation.	(47–49)
Reader proteins	HP1 proteins	Recognise H3K9me3 through chromodomains and contribute to heterochromatin formation and transcriptional silencing	(26, 50)
Writer: lysine methyltransferases	SUV39H1, SUV39H2, EZH2 and SETD2	Use S-adenosylmethionine as a methyl donor to methylate lysine residues. SUV39H enzymes catalyse H3K9 methylation; EZH2 catalyses H3K27 trimethylation within polycomb repressive complex 2; and SETD2 catalyses H3K36 trimethylation.	(29, 39, 40)

CARM1, coactivator-associated arginine methyltransferase 1; DOT1L, disruptor of telomeric silencing 1-like; EZH2, enhancer of zeste homolog 2; HP1, heterochromatin protein 1; JMJD6, Jumonji domain-containing protein 6; KDM, lysine demethylase; KMT, lysine methyltransferase; LSD, lysine-specific demethylase; PADI4/PAD4, peptidyl arginine deiminase 4; PRMT, protein arginine methyltransferase; SETD2, SET domain-containing protein 2; SUV39H, suppressor of variegation 3–9 homolog.

The table summarizes representative histone methylation-related enzymes and reader proteins based on established chromatin biology; inclusion in the table does not imply direct evidence of involvement in diabetic foot ulcer pathology.

#### 2.2.3 Histone phosphorylation

Histone phosphorylation occurs primarily on serine, threonine and tyrosine residues and is a rapid, dynamic and highly reversible histone post-translational modification (PTM). Similar to histone methylation, its effects on chromatin structure and gene expression depend strongly on the modified site and cellular context. Distinct phosphorylation sites participate in diverse processes, including transcriptional regulation, chromosome condensation, DNA damage repair, cell-cycle progression, apoptosis and stress responses. For example, H3S10ph and H3S28ph participate in mitotic chromosome condensation and, in selected contexts, stimulus-induced transcriptional responses (51, 52). By contrast, phosphorylation of H2AX at serine 139 (γ-H2AX) is an important marker of the DNA-damage response to DNA double-strand breaks (53–55). Protein kinases and phosphatases coordinate the regulation of histone phosphorylation. Histone phosphorylation-related enzymes and reader proteins supported by current evidence are summarized in **Table 4**.

**Table 4:** Representative enzymes and reader proteins involved in histone phosphorylation.

Functional category	Representative enzymes/proteins	Key functional features	References
Kinases	MSK1 and MSK2	Activated downstream of ERK and p38 MAPK signalling and catalyse histone H3 phosphorylation, particularly H3S10ph, in stimulus-responsive transcriptional programmes.	(56, 57)
	RSK2	Participates in mitogen-induced histone H3 phosphorylation and links extracellular growth-factor signalling to chromatin regulation.	(58)
	IKKα	Mediates H3S10 phosphorylation in response to cytokine stimulation and contributes to the activation of selected NF-κB-responsive transcriptional programmes.	(59)
	Aurora B	Functions primarily during mitosis and contributes to histone H3 phosphorylation, chromosome condensation and progression through cell division.	(51, 52, 60)
	DNA-damage-activated kinases, including ATM, ATR and DNA-PK	Participate in the phosphorylation of H2AX at serine 139 following DNA double-strand breaks, contributing to the formation of γ-H2AX and activation of the DNA-damage response. Their relative contributions may vary according to the cellular context and type of DNA damage.	(53–55)
	MST1	Catalyses phosphorylation of H2B at serine 14, a modification associated with chromatin condensation and apoptotic chromatin changes.	(61)
Phosphatases	PP1	Participates in the removal or regulation of mitosis-associated histone phosphorylation marks and contributes to the control of chromatin events during cell division.	(60)
	PP4	Dephosphorylates γ-H2AX and contributes to termination of DNA-damage signalling and recovery from DNA-damage checkpoints.	(62, 63)
Reader proteins	MDC1 containing BRCT domains	Recognises phosphorylated H2AX through BRCT domains and acts as a scaffold for the recruitment and assembly of DNA-damage response factors.	(55)
	14-3-3 proteins	Recognise phosphorylated and phosphoacetylated histone H3 and may cooperate with adjacent modifications, including H3K14ac, to regulate stimulus-induced transcription.	(64)
Kinases	MSK1 and MSK2	Activated downstream of ERK and p38 MAPK signalling and catalyse histone H3 phosphorylation, particularly H3S10ph, in stimulus-responsive transcriptional programmes.	(56, 57)

ATM, ataxia-telangiectasia mutated; ATR, ataxia-telangiectasia and Rad3-related protein; BRCT, BRCA1 C-terminal; DNA-PK, DNA-dependent protein kinase; ERK, extracellular signal-regulated kinase; H2AX, H2A histone family member X; H3S10ph, histone H3 serine 10 phosphorylation; H3S28ph, histone H3 serine 28 phosphorylation; H3K14ac, histone H3 lysine 14 acetylation; IKKα, inhibitor of nuclear factor kappa B kinase subunit alpha; MDC1, mediator of DNA-damage checkpoint 1; MST1, mammalian sterile 20-like kinase 1; MSK1/2, mitogen- and stress-activated protein kinase 1/2; NF-κB, nuclear factor kappa B; PP1, protein phosphatase 1; PP4, protein phosphatase 4; RSK2, ribosomal S6 kinase 2; γ-H2AX, phosphorylated H2AX at serine 139.

The table summarizes representative components of histone phosphorylation regulation based on established chromatin and DNA-damage biology. The inclusion of an enzyme or reader protein does not imply direct evidence of its involvement in diabetic foot ulcer pathology. The effects of histone phosphorylation are modification-site-, cell-type- and context-dependent.

#### 2.2.4 Other histone modifications, including ubiquitination, sumoylation, citrullination, and lactylation

Beyond acetylation, methylation and phosphorylation, histones are subject to several additional post-translational modifications, including ubiquitination, lactylation, SUMOylation and citrullination. Although the extent to which these modifications have been investigated, and the direct evidence supporting their roles in chronic wounds, vary, they collectively constitute a complex, dynamic and reversible chromatin-regulatory network. Histone ubiquitination contributes to the regulation of chromatin structure, DNA damage responses and transcription and may act in concert with other histone modifications to influence gene expression (65, 66). Histone SUMOylation is generally associated with transcriptional repression and regulation of chromatin states, although its effects may be context dependent. It can also engage in functional crosstalk with other modifications, including acetylation and ubiquitination (67, 68). Histone lactylation is a recently identified metabolism-associated modification that links lactate availability to transcriptional regulation (69, 70). In neutrophils, PAD4-mediated histone citrullination can promote chromatin decondensation and facilitate neutrophil extracellular trap (NET) formation (48, 49). However, the temporal dynamics, cell-type specificity and causal relevance of histone lactylation and citrullination/NET-associated pathways in human DFU tissue remain insufficiently established. Detailed evidence and unresolved questions are discussed in Sections 3.1.4 and 3.1.5. Collectively, these additional histone modifications provide a framework for understanding the links among chronic inflammation, metabolic abnormalities and impaired tissue repair from the perspective of histone post-translational modifications (hPTMs).

### 2.3 Mechanisms by which histone modifications and their regulatory enzymes control wound-healing-related gene expression

#### 2.3.1 Regulation of inflammatory cytokine expression

The spatiotemporal expression of inflammatory cytokines is critical for the orderly transition from the inflammatory to the proliferative phase of wound healing. Under physiological conditions, the early inflammatory response to tissue injury facilitates the clearance of pathogens and necrotic tissue. By contrast, sustained overexpression of pro-inflammatory cytokines can prolong neutrophil and macrophage infiltration, exacerbate tissue damage and impair the initiation of reparative program. Increasing evidence indicates that histone modifications regulate the transcription of multiple inflammatory mediators by modulating chromatin accessibility at the promoters and enhancers of inflammation-related genes. These modifications therefore represent an important epigenetic mechanism linking injury-associated signals to inflammatory transcriptional program (8, 28, 71).

The effects of different deacetylases on inflammatory transcription are highly context-dependent. Chen et al. found that Hdac3 deficiency substantially impaired the activation of the macrophage inflammatory gene-expression program, affecting a large subset of inflammation-associated genes. This effect was associated with reduced IFN-β expression, impaired STAT1-dependent transcription, and increased acetylation at nuclear receptor-associated gene loci (71). These findings suggest that HDAC3 is involved not only in transcriptional repression but may also support the proper activation of inflammatory gene program by maintaining specific signaling pathways and chromatin states. By contrast, Yeung et al. reported that SIRT1 can inhibit the transcriptional activity of NF-κB RelA/p65 by deacetylating its K310 residue (72). Because p65 is a non-histone substrate, this finding indicates that the effects of deacetylation on inflammation involve both chromatin state and transcription-factor activity. Collectively, these studies suggest that deacetylation may coordinate chromatin state, the expression of signaling molecules, and transcription-factor activity to shape the dynamic regulation of inflammatory gene-expression program and contribute to the transition from inflammation to tissue repair. However, whether histone and non-histone substrates are subject to coordinated regulation and whether regulatory axes mediated by different deacetylases form an interconnected network remain to be elucidated.

In addition to acetylation, histone phosphorylation may contribute to the rapid regulation of inflammatory cytokine transcription. Yamamoto et al. reported that IKKα mediates histone H3 serine 10 phosphorylation (H3S10ph) and promotes cytokine-induced gene expression. These findings suggest that histone phosphorylation not only contributes to chromatin remodeling but may also amplify inflammatory signaling, thereby facilitating the rapid activation of pro-inflammatory genes (59). Although this mechanism may support the clearance of pathogens and necrotic tissue during the early stage of wound repair, its persistent activation may sustain a local pro-inflammatory microenvironment and impede the transition from the inflammatory to the proliferative phase of wound healing.

The transcriptional effects of histone methylation depend strongly on the modified residue, genomic location and cellular context. H3K4me3 is generally associated with transcriptionally active chromatin, whereas H3K9me3 and H3K27me3 are more commonly linked to chromatin compaction and transcriptional repression. Cao et al. showed that EZH2, the catalytic subunit of the PRC2 complex, catalyzes the formation of H3K27me3 and contributes to Polycomb-mediated gene silencing by establishing a repressive chromatin environment (40). H3K27me3 may therefore function not only as a correlate of transcriptional repression but also as part of a mechanism that stabilizes silenced chromatin through the recruitment of downstream repressive complexes. In diabetic wounds, dysregulated deposition or removal of H3K27me3 at inflammation- or repair-related loci could potentially alter the transcriptional responsiveness of relevant genes. However, the direct roles of this modification in diabetic wound inflammation and tissue repair require validation in appropriate cell types and disease models.

#### 2.3.2 Regulation of genes involved in angiogenesis

Angiogenesis is an essential component of wound repair. The ingrowth of newly formed blood vessels delivers oxygen and nutrients to cells within the wound bed and provides a supportive microenvironment for reparative cell migration and extracellular matrix (ECM) deposition. Mechanistically, angiogenesis depends primarily on the proliferation, migration, lumen formation, maturation and remodeling of vascular endothelial cells in response to extracellular cues. Recent studies indicate that, in addition to canonical signaling pathways, including VEGF–VEGFR, Notch and Ang-1–Tie2 signaling, histone modification-mediated changes in chromatin state provide an additional layer of regulation for angiogenesis-related genes.

With respect to histone acetylation, angiogenic signals can promote blood vessel formation by regulating the transcription of key angiogenesis-related genes in endothelial cells. For example, Dutta et al. reported that angiogenic signals induce HIRA expression in endothelial cells. HIRA, in turn, promotes transcriptional induction of VEGFR1 and other angiogenesis-related genes by mediating the incorporation of H3.3K56ac into chromatin at these loci. HIRA depletion reduces H3.3K56ac deposition at these loci and suppresses the expression of VEGFR1 and other angiogenesis-related genes, together with endothelial network formation and angiogenesis *in vivo (*73);Yan et al. found that the histone acetyltransferase KAT7 maintains VEGFR2 expression and the angiogenic capacity of endothelial cells by regulating H3K14 and H4 acetylation, as well as RNA polymerase II occupancy, within the VEGFR2 gene body. KAT7 depletion reduced VEGFR2 expression and impaired endothelial angiogenesis (74). Meanwhile, Shu et al. found that VEGF enhances the expression of genes involved in cell–cell adhesion by inducing H3/H4 acetylation at the VE-cadherin promoter (75). VEGF may also influence transcription of hMT1G and VEGFA by inhibiting HDAC1 or modulating SIRT2 activity (76, 77). Collectively, the available evidence indicates that histone acetylation regulates multiple components of VEGF–VEGFR signaling, including receptor expression, endothelial cell–cell junction integrity and transcription of downstream effector genes. Through these coordinated effects, histone acetylation may modulate endothelial responsiveness to angiogenic stimuli and promote vascular network formation.

With respect to histone methylation, this modification may contribute to hypoxic adaptation, angiogenesis and vascular maturation by regulating the transcriptional states of angiogenesis-related genes under hypoxic conditions. For example, hypoxia–HIF signaling may promote the expression of MMP12 and other genes involved in vascular remodeling through KDM3A-mediated alterations in H3K9 methylation, thereby enhancing cellular invasive capacity and contributing to vascular structural remodeling. KDM3A knockdown suppresses hypoxia–HIF-dependent gene transcription and attenuates the associated invasive and vascular-remodeling effects. In placental trophoblasts, the hypoxia–HIF–KDM3A–MMP12 axis regulates trophoblast plasticity and hypoxia-associated invasive behavior. This study provides indirect mechanistic context for hypoxia-responsive chromatin regulation and tissue remodeling, but it should not be interpreted as direct evidence for endothelial angiogenesis or vascular repair in diabetic wounds. Whether this axis operates in wound endothelial cells or other DFU-associated vascular compartments remains unknown (78). EZH2, a histone H3 lysine 27 methyltransferase, also participates in the regulation of VEGF-mediated angiogenesis in endothelial cells. Studies have shown that VEGF downregulates miR-101, thereby relieving miR-101-mediated repression of EZH2 and enhancing EZH2-dependent H3K27 methylation and associated transcriptional program. This process promotes endothelial cell sprouting and migration. Conversely, miR-101 upregulation, EZH2 knockdown or treatment with the EZH2 inhibitor DZNep suppresses these angiogenic phenotypes (79). Overall, histone methylation may coordinate hypoxic responses, angiogenesis and vascular maturation by balancing transcriptionally active and repressive states.

#### 2.3.3 Regulation of cell proliferation, migration, and differentiation

During wound healing, cells must rapidly respond to extracellular and intracellular cues that regulate proliferation, migration, differentiation and apoptosis, thereby contributing to tissue repair. For example, the mitogen-activated protein kinase (MAPK) pathway regulates the expression of genes involved in cell proliferation, migration and differentiation. In addition to directly modulating transcription-factor activity through phosphorylation, MAPK signaling may also contribute to epigenetic regulation by influencing chromatin structure (80). MSK1 and MSK2 provide an example of how MAPK signaling may regulate histone modifications. Dunn and Davie proposed that activation of ERK and p38 MAPK promotes the phosphorylation of histone H3 at serine 28 (H3S28ph). Lau et al. subsequently proposed that H3S28ph may weaken the association of Polycomb group (PcG) complexes with target gene promoters and reduce the enrichment of the repressive mark H3K27me3, thereby relieving transcriptional repression and facilitating gene activation (81). Although the extent to which this mechanism operates in specific wound-healing cell types remains to be established, these findings provide a potential link between extracellular signaling and the dynamic regulation of chromatin states.

Collectively, the available evidence indicates that post-translational modifications, including histone acetylation, methylation and phosphorylation, may contribute to wound-repair processes such as inflammatory resolution, angiogenesis, granulation-tissue formation and tissue remodeling. These modifications can influence the chromatin states of genes involved in inflammatory responses, angiogenesis, and cellular proliferation, migration and differentiation. Histone modifications therefore represent an important regulatory interface between injury-associated signals and cellular transcriptional responses. Their dynamic regulation may help coordinate the transitions between successive phases of wound repair. In diabetes, however, this tightly regulated epigenetic network may be disrupted by a persistent pathological microenvironment, potentially contributing to defective cell responses and delayed tissue repair.

## 3 Mechanisms by which histone modifications contribute to impaired diabetic wound healing

### 3.1 Impaired resolution of inflammation and dysregulated macrophage polarization

#### 3.1.1 Persistent inflammation and dysregulated macrophage polarization in diabetic wounds

In diabetic wounds, the temporal dynamics of inflammatory cell recruitment and macrophage phenotypic transition may be disrupted. This dysregulation is characterized by the persistent predominance of pro-inflammatory M1-like macrophages and an insufficient reparative M2-like macrophage response (**Figure 1**) (These labels are used here as functional descriptors rather than as strictly discrete or terminal macrophage lineages). Previous studies have demonstrated that macrophage recruitment is closely associated with monocyte chemoattractant protein 1 (MCP-1) and its receptor, CCR2 (82). Kimball et al. reported that intrawound administration of an MCP-1-neutralising antibody in diabetic mice reduced the accumulation of M1-like macrophages and promoted wound healing (83).

The canonical NF-κB signaling pathway is an important contributor to M1-like macrophage polarization. Single-cell RNA sequencing analyses by Davis et al. demonstrated that NF-κB-mediated inflammatory activity was markedly increased in diabetic wounds and was associated with elevated expression of cyclooxygenase 2 (COX2) and prostaglandin E2 (PGE2). Genetic manipulation or macrophage-targeted COX2 nanotherapy aimed at inhibiting this inflammatory axis reportedly shifted macrophages away from an M1-like phenotype and improved wound healing (84). Similarly, studies have suggested that inhibition of excessive kallikrein-binding protein (KBP) activity in diabetic wounds can suppress NF-κB signaling, reduce M1-like macrophage polarization and improve wound repair (85). Overall, impaired transition away from the M1-like macrophage state and persistent inflammation at the wound site are key contributors to impaired healing in diabetic wounds. Therefore, promoting the resolution of macrophage-associated inflammation may facilitate progression to the proliferative phase of wound healing.

#### 3.1.2 Histone methylation remodeling of macrophage inflammatory programs

However, how transient activation of inflammatory signaling is converted into a persistent transcriptional state requires further clarification at the level of histone post-translational modifications (hPTMs). Evidence from diabetic wound models suggests that histone methylation-related enzymes may contribute to the maintenance and amplification of macrophage inflammatory program by altering chromatin states at inflammatory gene loci.

MLL1, a methyltransferase that promotes H3K4me3 deposition, may enhance TLR4 expression by increasing H3K4me3 enrichment at the TLR4 promoter in diabetic macrophages (86, 87). Consistent with this observation, Kimball et al. used a myeloid-specific MLL1-knockout model and found that MLL1 increased H3K4me3 enrichment near NF-κB-binding sites at the promoters of inflammatory genes in macrophages. Genetic depletion of MLL1 reduced the expression of pro-inflammatory cytokines and improved wound healing, supporting a role for the MLL1–H3K4me3 axis in diabetic wound inflammation (88).

By contrast, SETDB2 catalyzes the formation of the repressive histone mark H3K9me3. Evidence from diabetic wound models indicates that SETDB2 deficiency is associated with increased chromatin accessibility at NF-κB-dependent inflammatory loci in macrophages. By maintaining a repressive chromatin state at selected regulatory regions, SETDB2 may restrict transcription-factor access to inflammatory genes. Accordingly, reduced SETDB2 expression or function may be involved in persistent macrophage inflammation and impaired wound repair (89). However, the precise genomic recruitment mechanisms underlying this effect, as well as their relevance to human DFU tissue, remain incompletely defined.

Building on these findings, Mangum et al. reported that SETDB2 deficiency increased chromatin accessibility at the promoters of NF-κB-dependent inflammatory genes and enhanced inflammatory transcriptional program. These findings support the view that SETDB2 may restrain NF-κB-mediated inflammatory responses by remodeling chromatin states at regulatory regions of inflammatory genes. The study further showed that STAT3 is required to maintain SETDB2 expression in macrophages and can interact with SETDB2 while inhibiting its interaction with the NF-κB subunit RELA. This interaction may weaken the ability of SETDB2 to restrain NF-κB-driven inflammatory transcription. In macrophages from diabetic wounds, both STAT3 expression and STAT3–SETDB2 binding were increased, suggesting that STAT3 could help explain impaired SETDB2-mediated anti-inflammatory activity and persistence of the macrophage inflammatory phenotype by regulating SETDB2 expression and its functional interaction with RELA (90).

In contrast to SETDB2, which may restrain inflammatory transcription through H3K9me3 deposition, JMJD3 may promote NF-κB-dependent inflammatory gene expression by removing the repressive H3K27me3 mark from selected gene promoters. Davis et al. found that palmitate (C16:0), a saturated fatty acid associated with type 2 diabetes, upregulated JMJD3 expression in monocyte-derived macrophages through TLR4–MyD88-dependent signaling. JMJD3-mediated removal of H3K27me3 from NF-κB-regulated inflammatory loci was associated with increased inflammatory cytokine transcription and persistence of the pro-inflammatory macrophage state (91, 92).

Overall, the MLL1–H3K4me3, SETDB2–H3K9me3 and JMJD3–H3K27me3 axes represent distinct but potentially convergent mechanisms involved in macrophage inflammatory program in diabetic wounds. MLL1-associated H3K4me3 enrichment may promote transcriptionally active states, whereas SETDB2-associated H3K9me3 may restrain inflammatory gene expression. By contrast, JMJD3-mediated removal of H3K27me3 may relieve transcriptional repression at selected inflammatory loci. These pathways may converge on NF-κB- and STAT3-associated transcriptional networks. Nevertheless, their temporal relationships, cell-type specificity and relevance to human DFU remain to be determined.

#### 3.1.3 Dysregulated histone acetylation sustains inflammation in diabetic wounds

Dysregulation of histone acetylation and deacetylation may also contribute to persistent inflammation in diabetic wounds. Evidence for this possibility ranges from human DFU-associated observations to mechanistic and intervention-based studies in diabetic wound models and macrophage systems. MOF (males absent on the first), also known as KAT8, is a histone acetyltransferase whose principal histone substrate is histone H4 lysine 16 (H4K16). Evidence indicates that MOF expression is elevated in macrophages from diabetic mouse wounds, accompanied by increased H4K16ac enrichment at the promoters of inflammatory genes and enhanced expression of inflammatory cytokines. Conversely, myeloid cell-specific deletion of MOF reduces the expression of inflammation-related genes, suggesting that MOF may promote the TNF-α–NF-κB inflammatory transcriptional program through increased H4K16 acetylation. In addition, TNF-α induces MOF expression, whereas etanercept, a TNF-α inhibitor, reduces MOF levels and improves wound healing in obese mice. These findings suggest that TNF-α and MOF may form a feedback relationship that sustains inflammation (93). Sirtuin-mediated deacetylation may likewise regulate persistent inflammation in diabetic wounds. In a 2020 clinical study, Teena et al. reported that patients with diabetic foot ulcers exhibited reduced SIRT6 expression, increased levels of pro-inflammatory cytokines, including IL-6 and TNF-α, and decreased levels of IL-10 (94). Subsequently, in 2024, Xue et al. demonstrated *in vivo* that SIRT6 deficiency impaired repair of diabetic ulcers. This impairment was accompanied by enhanced F4/80- and NF-κB p50-associated signals, increased expression of inflammatory mediators, including IL-1β, TNF-α and IL-6, and dysregulated expression of repair-associated factors such as IL-4 and IL-10 (95). However, this study did not examine specific histone acetylation sites or determine whether the effects of SIRT6 occurred predominantly in macrophages. Therefore, these findings alone are insufficient to establish that SIRT6 regulates wound inflammation primarily through histone deacetylation.

In 2026, Chen et al. provided mechanistic evidence relevant to this question. They reported that SIRT6 levels were reduced in M1-like macrophages under diabetic wound conditions and following inflammatory stimulation. This reduction was accompanied by lysine hyperacetylation, increased acetylation of the non-histone substrate p65 at Lys310, and elevated histone H3K9ac levels. Activation or stabilization of SIRT6 promoted deacetylation of p65 Lys310 and H3K9ac, thereby suppressing p65-dependent inflammatory transcription and M1-like macrophage polarization. Pharmacological inhibition of SIRT6 partially reversed these effects (96).

Recent diabetic wound-model evidence further implicates the p300/CBP acetyltransferase–ZEB2 axis in macrophage dysfunction. Roy et al. reported that hyperglycemic conditions increased p300/CBP acetyltransferase activity and promoted ZEB2-associated pro-inflammatory macrophage responses, thereby delaying diabetic wound healing (97). Pharmacological inhibition of p300/CBP activity reduced ZEB2 expression or activity and attenuated inflammatory macrophage program in diabetic wound models. These findings provide direct intervention-based evidence that excessive acetyltransferase activity may contribute to the persistence of macrophage inflammation in diabetic wounds. However, because p300/CBP modifies multiple histone and non-histone substrates, the extent to which the observed effects are mediated specifically through a defined histone acetylation site remains to be established.

A complementary study showed that restoration of histone acetylation can improve the temporal organization of macrophage responses during diabetic wound repair. Singh et al. reported that butyrate-based treatment restored histone acetylation, including H3K27ac-associated regulatory activity, and improved the spatiotemporal dynamics of macrophage responses in diabetic wounds (98). This intervention was associated with reduced persistence of inflammatory macrophage program and improved coordination between macrophages and reparative tissue cells, ultimately accelerating diabetic wound repair. These findings provide direct intervention-based evidence that acetylation-related chromatin states are not merely associated with diabetic wound inflammation but can be therapeutically modulated in a diabetic wound model. However, the responsible histone sites, genomic loci, macrophage subpopulations and treatment windows remain incompletely defined. In addition, butyrate may alter metabolism, non-histone protein acetylation and other pathways, so the contribution of H3K27ac to the observed healing benefit cannot yet be considered exclusive or causally sufficient.

Taken together, current diabetic wound-model studies identify several acetylation-related regulatory patterns in macrophages: MOF-associated H4K16 acetylation and p300/CBP-associated signaling may promote inflammatory transcription, whereas SIRT6-mediated deacetylation may restrain p65-dependent inflammatory activity. Conversely, restoration of histone acetylation, including H3K27ac-associated regulatory activity, may improve the spatiotemporal organization of macrophage responses and promote tissue repair. These findings provide direct preclinical evidence that acetylation-related chromatin regulation is functionally involved in diabetic wound inflammation and repair. Nevertheless, the effects of these regulators are unlikely to be determined by histone substrates alone, and their therapeutic interpretation remains limited by incomplete information regarding locus specificity, causal macrophage populations, stage-dependent effects, non-histone substrates and relevance to human DFUs.

#### 3.1.4 Histone lactylation: a putative metabolic–epigenetic regulator of inflammation resolution in diabetic wounds

Histone lactylation has emerged as a potential mechanism through which cellular metabolism may influence inflammatory gene expression. Current evidence combines mechanistic findings from *in vitro* macrophage systems with direct intervention-based evidence from diabetic wound models. However, the latter does not yet establish a histone-lactylation-specific causal mechanism. Zhang et al. identified histone lysine lactylation (Kla) during macrophage activation and proposed that intracellular lactate accumulation during the later stages of LPS- and IFN-γ-induced macrophage activation is associated with increased Kla at loci linked to tissue-repair-associated genes (69). In this experimental setting, histone lactylation was proposed to contribute to the transition from an inflammatory macrophage program towards a more reparative transcriptional state. However, these observations were generated primarily in *in vitro* macrophage systems and should not be interpreted as evidence that an identical mechanism has been established in diabetic wound tissue.

Macrophages in diabetic wounds are exposed to hyperglycemia, hypoxia, oxidative stress, altered lipid metabolism and persistent inflammatory stimuli, all of which may affect glycolytic activity and lactate handling. These conditions could theoretically alter the availability of lactate or lactate-derived acyl donors and thereby influence histone lactylation dynamics. Nevertheless, the direction and functional consequences of this relationship remain uncertain. Lactate accumulation may support selected reparative transcriptional program under some conditions, whereas persistent metabolic stress may instead be associated with dysregulated or spatially restricted lactylation responses. Thus, the hypothesis that sustained lactate accumulation under diabetic conditions persistently remodels histone lactylation and blocks inflammation resolution remains biologically plausible but has not been fully validated.

More recent evidence from a diabetic wound model provides direct intervention-based support for a role of lactate metabolism in this process. Wu et al. developed a lactate-oxidizing single-atom nanozyme that promoted lactate oxidation, altered the metabolically stressed wound microenvironment, reduced lactylation-related signals and accelerated diabetic wound healing (99). These findings indicate that manipulation of lactate metabolism can influence lactylation-associated responses in diabetic wounds and provide proof-of-concept evidence for a metabolic–epigenetic therapeutic strategy. However, the study did not establish that histone lactylation at defined genomic loci is necessary or sufficient for the healing effect. Lactate oxidation may also alter oxidative metabolism, mitochondrial activity, reactive oxygen species and other inflammatory pathways. Moreover, the responsive cell populations, the specific histone Kla species involved and the temporal window during which lactylation is therapeutically beneficial remain unresolved.

Direct evidence from human DFU biopsies remains particularly limited. It has not yet been determined whether lactate concentrations, histone Kla abundance, locus-specific Kla occupancy and macrophage functional states are coordinately altered in human DFU tissue. It is also unclear whether histone lactylation differs between the wound edge, wound bed and adjacent non-ulcerated skin, or whether its temporal dynamics predict healing, infection, recurrence or response to treatment. Addressing these questions will require longitudinal human DFU biopsy studies integrating local metabolite measurements with cell-type-resolved transcriptomic and epigenomic analyses, including histone Kla profiling in macrophages and other wound-resident cells. Accordingly, histone lactylation should be regarded as a candidate metabolic–epigenetic mechanism supported by direct preclinical evidence of lactate-metabolism-dependent modulation in diabetic wounds, but not yet as an established, locus-specific driver of impaired inflammation resolution in human DFUs.

#### 3.1.5 Histone citrullination, NETosis and unresolved questions in DFU

Histone citrullination and NET formation represent another potentially relevant but incompletely characterized pathway in diabetic wound healing. PAD4-mediated citrullination of histones, particularly histone H3, promotes chromatin decondensation and facilitates the extrusion of chromatin-containing NETs by activated neutrophils (48, 49). NETs can contribute to antimicrobial defense; however, excessive or persistent NET formation may amplify tissue injury, impair cell migration and prolong inflammatory signaling.

Experimental evidence indicates that diabetes can prime neutrophils towards NETosis and delay wound healing (100). In addition, Fadini et al. reported enrichment of NET-related components in non-healing human DFUs, increased circulating NET-associated proteins in patients with DFUs, and enhanced spontaneous NETosis in neutrophils isolated from these patients (101).

These observations provide clinically relevant support for an association between NET-related activity and poor healing. However, they do not establish a direct causal pathway in human DFU tissue from PAD4 activation to histone citrullination, NET release, persistent inflammation and impaired healing.

Several questions therefore remain unresolved. First, it is unclear whether citrullinated histones are persistently increased in serial human DFU biopsies and whether their abundance differs between infected and non-infected ulcers or between healing and non-healing wounds. Second, the cellular and spatial distribution of PAD4 activity, citrullinated histones and NET structures within the wound bed has not been adequately defined. Third, it remains uncertain whether NETosis is a primary driver of chronic inflammation, a consequence of infection and tissue damage, or both. Finally, because NET formation also contributes to host defense, excessive inhibition of PAD4 or NETosis could theoretically compromise antimicrobial protection in infected wounds.

Accordingly, histone citrullination and NETosis should be regarded as mechanistically plausible contributors to impaired DFU healing, but their causal role and therapeutic tractability require validation in well-characterized human wound tissue. Future studies should combine spatial detection of citrullinated histone H3 and NET components with microbiological assessment, inflammatory profiling and longitudinal clinical outcomes.

### 3.2 Histone modifications and impaired angiogenesis

#### 3.2.1 Regulation of endothelial repair-related genes by histone acetylation and deacetylation

Available evidence ranges from associative observations in human DFUs to intervention-based studies in diabetic wound models and indirect mechanistic findings from vascular or endothelial systems. Histone acetylation may help maintain the VEGF–VEGFR-associated angiogenic program. Available evidence suggests that aberrant expression of HDAC and sirtuin family members in diabetic foot ulcers is associated with dysregulation of Nrf2- and VEGF-related angiogenic signaling. In a study of wound tissues from patients with diabetic foot ulcers of different grades, expression of Nrf2 and the associated pro-angiogenic factors VEGF, HIF-1α and SDF-1α progressively decreased with increasing ulcer severity, whereas expression of the angiogenesis inhibitor thrombospondin-2 (TSP-2) increased. Meanwhile, expression of HDAC1, HDAC3, HDAC4 and HDAC11, as well as SIRT3, SIRT5, SIRT6 and SIRT7, was increased, whereas that of HDAC2, HDAC8, SIRT1, SIRT2 and SIRT4 was decreased. Among these factors, HDAC4 expression was significantly negatively correlated with Nrf2 and several angiogenic markers (102). The abnormal expression pattern represented by increased HDAC4 levels suggests that diabetic wounds may be subject to unfavorable regulation of Nrf2- and VEGF-related pro-angiogenic gene transcription. By contrast, the role of SIRT6 in diabetic animal wounds is supported by interventional evidence. Rajarajan et al. found that SIRT6 knockdown at wound margins in an animal model delayed wound closure, reduced the density of CD31-positive capillaries and suppressed VEGF expression in wound tissue (103). In addition to its effects on VEGF-related pro-angiogenic signaling, a review by Beegum et al. summarized evidence suggesting that increased SIRT6 expression may reduce oxidative stress, inflammation and apoptosis in vascular endothelial cells and restore endothelial migration and tube formation under high-glucose conditions (104). In other words, abnormal histone-modification patterns may act at both the level of insufficient angiogenic signaling and that of impaired endothelial responsiveness, making it difficult for diabetic wounds to establish a stable and effective microvascular network. Because inadequate neovascularization further restricts oxygen and nutrient delivery and affects granulation-tissue maturation and the function of other reparative cells, dysregulation of histone deacetylation may represent an important regulatory link between the diabetic metabolic environment and wound-healing failure. Nevertheless, the precise roles of individual HDAC and Sirtuin subtypes in this process may depend on cell type, disease stage, and their specific chromatin targets. Current studies of HDAC4 mainly demonstrate an association with expression patterns, whereas the role of SIRT6 is supported by some animal and cellular intervention evidence; the strength of evidence for these two factors is therefore not equivalent. Future studies should further define the direct target genes, temporal effects, and cell-type specificity of individual subtypes, and determine whether targeting histone deacetylation can improve angiogenesis while promoting overall healing of diabetic wounds.

#### 3.2.2 Putative histone-based metabolic memory in diabetic vascular disease

In addition to impaired wound repair, metabolic memory is a widely recognized feature of diabetic vascular complications. Molecular imprints established during prior exposure to hyperglycemia may allow vascular injury to persist or progress despite subsequent partial improvement in glycemic control (105, 106). Although metabolic memory has been linked to stable or semi-stable epigenetic alterations, most evidence implicating histone modifications has been derived from diabetic vascular tissues, vascular smooth muscle cells, or experimental models of vascular injury. Direct evidence demonstrating persistent histone methylation abnormalities in human DFU wound tissue remains scarce. Therefore, the studies discussed below should be interpreted primarily as indirect mechanistic evidence from diabetic vascular disease that may provide insights into DFU-associated vascular dysfunction, rather than as definitive evidence that histone modification-related metabolic memory is present in human DFU.

Within the H3K9 methylation pathway, Villeneuve and colleagues used vascular smooth muscle cells (VSMCs) isolated from db/db mice to investigate the relationship between H3K9 methylation and inflammatory activation. They found that H3K9me3 levels and *Suv39h*1 occupancy at the promoters of inflammation-related genes were significantly reduced, accompanied by decreased *Suv39h1* protein expression. Further analyses showed that miR-125b was upregulated in diabetic VSMCs and directly targeted the 3′ untranslated region (3′UTR) of *Suv39h1*, thereby suppressing *Suv39h1* protein expression (107).Moreover, *Suv39h1* overexpression partially reversed the pro-inflammatory phenotype of VSMCs derived from db/db mice, whereas *Suv39h1* knockdown in normal VSMCs induced the expression of inflammatory genes. Transfection with a miR-125b mimic further reduced H3K9me3 levels at the promoters of inflammation-related genes and increased the expression of inflammatory mediators, including MCP-1 and IL-6; inhibition of miR-125b produced the opposite effects (108). In contrast to the *Suv39h1*–H3K9me3 axis, KDM3A primarily catalyzes the demethylation of H3K9me1/2. In a separate study of diabetic vascular remodeling, Chen et al. reported that KDM3A expression was increased in diabetic blood vessels and was accompanied by persistent loss of H3K9me2. This alteration persisted during high-glucose exposure and after withdrawal of the high-glucose stimulus, suggesting a potential association with diabetes-related epigenetic memory. *In vitro* and *in vivo* experiments showed that KDM3A overexpression enhanced high-glucose- and angiotensin II (Ang II)-induced VSMC proliferation and migration and accelerated neointimal formation after balloon injury in diabetic rats without glycemic-lowering intervention. Conversely, KDM3A knockdown attenuated these changes. Mechanistically, KDM3A regulated the Rho–ROCK and Ang II–AGTR1 signaling pathways by modulating H3K9me2 levels at the proximal promoter regions of AGTR1 and ROCK2, thereby promoting diabetic vascular remodeling (109). Overall, the *Suv39h1*–H3K9me3 and KDM3A–H3K9me2 axes represent complementary but functionally distinct patterns of H3K9 methylation dysregulation in diabetic vascular disease. The former is primarily associated with loss of the repressive mark H3K9me3 and consequent derepression of inflammatory genes, whereas the latter involves enhanced H3K9me2 demethylation and abnormal VSMC proliferation and migration. Together with abnormalities in HDAC- and sirtuin-regulated histone acetylation, these findings provide mechanistic support for a potential role of histone-modification dysregulation in diabetic vascular inflammation and remodeling.

Notably, in some diabetic vascular models, H3K9 methylation abnormalities persist after withdrawal of the high-glucose stimulus, suggesting that they may contribute to the formation and maintenance of hyperglycemic or metabolic memory. Nevertheless, several key questions regarding their relevance to DFU remain unresolved. It is currently unclear whether the histone-methylation changes observed in diabetic VSMCs are also present in endothelial cells, pericytes, fibroblasts, or immune cells within human DFU tissues. It has also not been directly demonstrated in human DFU samples whether these epigenetic marks persist after glycemic control is achieved. Furthermore, it remains to be determined whether H3K9 methylation changes are causal drivers of impaired wound perfusion and angiogenesis or secondary consequences of diabetes-associated vascular injury. Finally, the temporal sequence and dynamic relationships among vascular-wall epigenetic alterations, microvascular dysfunction, and wound healing remain poorly defined. Addressing these questions will require longitudinal human DFU samples or well-characterized clinical follow-up cohorts, together with cell-type-resolved profiling of histone modifications, chromatin-accessibility analyses, and assessment of the expression and activity of the relevant enzymes.

### 3.3 Impaired myofibroblast differentiation and extracellular matrix remodeling

#### 3.3.1 Direct suppression of the myofibroblast gene program by histone methylation

Fibroblasts contribute to wound healing by differentiating into myofibroblasts, which subsequently promote wound contraction and closure (110). However, the regulatory mechanisms governing fibroblast-to-myofibroblast differentiation during wound healing remain incompletely understood. Recent studies nevertheless suggest that histone post-translational modifications (hPTMs) may contribute to impaired fibroblast-to-myofibroblast differentiation in diabetic wounds.

Bauer et al. found that TNF-α upregulates SETDB2 through the JAK1/3–STAT3 signaling pathway. SETDB2 subsequently represses the expression of myofibroblast-associated genes in an H3K9me3-dependent manner, thereby impairing fibroblast-to-myofibroblast differentiation. In diabetic wounds, excessive activation of this pathway contributes to impaired healing, whereas targeting SETDB2 or the JAK1/3–STAT3 pathway promotes myofibroblast differentiation and facilitates wound repair (111). This study not only elucidated a molecular mechanism through which the TNF-α–STAT3–SETDB2 axis regulates myofibroblast-associated gene expression but also addressed an important gap in the understanding of fibroblast-to-myofibroblast differentiation, thereby identifying a potential therapeutic target for diabetic wound treatment. Importantly, SETDB2 exerts distinct and, in some respects, opposing effects in macrophages (Section 3.1.2) and fibroblasts. In macrophages, reduced SETDB2 expression leads to decreased H3K9me3 levels and derepression of inflammatory genes. By contrast, in fibroblasts, TNF-α-induced upregulation of SETDB2 promotes H3K9me3-mediated silencing of myofibroblast-associated genes. Thus, the same molecule may exert different pathological effects through opposite changes in expression across cell types, highlighting the cell-type specificity of histone modification-mediated regulation.

These opposing effects also suggest that the biological consequences of SETDB2 cannot be inferred solely from its catalytic activity or overall expression level. Rather, the functional output of an hPTM regulator is likely shaped by the pre-existing chromatin accessibility landscape, the lineage-specific enhancer repertoire and the transcription-factor partners available in each cell type (112–114).

Macrophages and fibroblasts differ in their lineage-defining regulatory elements, basal chromatin accessibility and inflammatory transcriptional states, which may influence the genomic regions at which SETDB2 acts and determine which gene networks are sensitive to SETDB2-mediated H3K9me3 deposition (112, 115–117).

In macrophages, SETDB2-associated H3K9me3 regulation may contribute to the repression of NF-κB-dependent inflammatory transcription, whereas in fibroblasts, TNF-α–JAK/STAT3 signaling may promote SETDB2-dependent repression of regulatory program required for fibroblast-to-myofibroblast transition (89, 90, 111, 117).

Single-cell and spatial epigenomic approaches, including single-cell ATAC-seq, single-cell CUT&Tag and integrated transcriptomic–epigenomic profiling, could help resolve cell-type-specific hPTM landscapes in human DFU tissues and determine how SETDB2-associated chromatin regulation varies across macrophages, fibroblasts and other wound-resident cell populations (118–120).

#### 3.3.2 Indirect effects of inflammation–fibrosis coupling on matrix deposition

A persistently inflammatory microenvironment may also impair extracellular matrix remodeling through indirect mechanisms. When macrophages fail to acquire reparative functions, the production or regulation of factors such as IGF-1 and TGF-β1 may be altered, thereby limiting myofibroblast formation and collagen synthesis. Meanwhile, increased MMP activity may accelerate collagen degradation, further disrupting matrix deposition and tissue reconstruction (121). These findings indicate that diabetic wounds exhibit not only an impaired transition from the inflammatory to the proliferative phase but also dysfunction of proliferative-phase cells. These abnormalities may result from the actions of multiple cytokines in the hyperglycemic environment and ultimately contribute to impaired wound healing.

### 3.4 Impaired re-epithelialization and keratinocyte dysfunction

Re-epithelialization is a key event during the proliferative phase of wound healing. In diabetic wounds, however, keratinocyte migration, proliferation and differentiation are markedly impaired. Evidence from epidermal-specific animal models and non-diabetic cutaneous repair systems provides indirect mechanistic support for a role of SETD2 in keratinocyte behavior. Whether this pathway is dysregulated in diabetic wounds remains unclear. SETD2 is the principal established mammalian histone methyltransferase responsible for catalyzing H3K36 trimethylation. Li et al. generated mice with epidermis-specific deletion of Setd2 and found that SETD2 depletion enhanced HaCaT cell migration and proliferation by activating AKT–mTOR signaling, thereby promoting wound healing (122). These findings suggest that the SETD2–H3K36me3 axis may contribute to the regulation of keratinocyte behavior. However, whether SETD2 is downregulated under diabetic conditions and the mechanisms involved remain to be elucidated.

During wound re-epithelialization, Zhang et al. proposed that a hyperglycemic environment increases c-Myc levels and activates *S100A6* transcription through the Wnt–β-catenin pathway, thereby inhibiting keratinocyte differentiation and impairing wound re-epithelialization (123). However, whether this pathway is directly regulated by histone modifications remains unclear and warrants further investigation. Findings from acute or non-diabetic cutaneous injury models suggest that some HDAC inhibitors may influence keratinocyte proliferation and progenitor-cell responses. However, these observations do not establish efficacy in diabetic wounds, and the relevant cell-specific mechanisms remain incompletely defined (124, 125).

## 4 Potential therapeutic strategies and translational perspectives

Diabetic wounds are characterized by persistent inflammation, impaired cell migration, inadequate angiogenesis and dysregulated extracellular matrix remodeling. Consequently, interventions directed at a single target may have limited capacity to correct pathological alterations that occur simultaneously across different stages of the repair process. Recent preclinical studies have therefore explored multilayered therapeutic strategies targeting dysregulated histone modifications and metabolically regulated epigenetic networks. These approaches include direct modulation of histone-modifying enzymes, indirect regulation of chromatin states through metabolic reprogramming, and the use of engineered delivery platforms, such as hydrogels, microneedles, nanoparticles and exosomes, to enable localized and temporally controlled intervention. The principal targets, mechanisms, therapeutic effects and translational considerations of these strategies are summarized in **Table 1**. To date, no hPTM-targeted intervention has established clinical efficacy in patients with DFUs. Available human DFU studies are predominantly observational, whereas therapeutic evidence is derived mainly from diabetic animal models, indirect wound-repair systems and external clinical studies in unrelated diseases.

Recent studies suggest that hPTM-related interventions may improve selected features of diabetic wound repair, but most evidence remains preclinical and model dependent. Their target specificity, pharmacokinetics, biodistribution and reproducibility require further evaluation.

### 4.1 Important translational limitations should nevertheless be considered

Epigenetic therapeutics frequently influence broad transcriptional program rather than a single disease-specific target. This raises concerns regarding off-target effects, dose-related toxicity and unintended alterations in non-wound tissues (30, 34, 126). These concerns may be particularly relevant to DFU because affected individuals commonly present with complex metabolic, vascular, inflammatory and infection-related abnormalities. Moreover, the functions of histone-modifying enzymes are strongly dependent on cell type, substrate, genomic location and disease stage. For example, SETDB2 limits inflammatory transcription in macrophages but represses myofibroblast-associated gene program in fibroblasts, illustrating why non-selective modulation may produce divergent effects across wound-resident cells (89, 90, 109). The same principle may apply to pharmacological inhibitors, whose effects can vary according to target expression, cellular context, dose and treatment duration. Therefore, systemic, non-selective or prolonged inhibition of an epigenetic regulator may not reproduce the beneficial effects observed with cell-targeted or temporally controlled intervention.

Therapeutic timing represents an additional challenge. Early inflammatory responses are required for pathogen clearance and removal of damaged tissue, whereas persistence of inflammatory program impedes the transition towards tissue repair. Excessive suppression of innate immune activation during the early phase could therefore compromise host defense, whereas limiting persistent inflammatory activation during later stages may facilitate inflammation resolution and tissue repair (17, 19, 98). Similarly, an intervention that is beneficial during the inflammatory phase may be inappropriate during the proliferative or remodeling phases (6, 19, 127). These considerations suggest that the dose, duration, cellular target and timing of epigenetic treatment should be tailored to the biological stage of the wound.

Another important limitation is that the therapeutic effects reported in experimental studies may not be attributable exclusively to the intended histone-modification pathway. Some compounds used in preclinical studies can affect non-histone proteins, multiple signaling pathways or cellular metabolism. For example, HDAC inhibitors may influence both histone acetylation and the acetylation or activity of non-histone transcription factors. Similarly, metabolic interventions may alter intracellular redox status, mitochondrial activity and inflammatory signaling in addition to modifying histone acylation. Thus, future studies should combine pharmacological intervention with genetic validation, locus-specific chromatin profiling and appropriate rescue experiments to establish target specificity.

### 4.2 Rationale for local and temporally controlled delivery

Local delivery is therefore likely to be particularly important for the clinical development of epigenetic therapies for DFU. Local administration may increase drug exposure within the wound bed while reducing systemic concentrations and limiting exposure of distant tissues. This approach may be especially advantageous for inhibitors of HDACs, histone methyltransferases, demethylases or other chromatin regulators that are expressed in multiple organs and participate in essential physiological processes.

Biomaterial-based platforms, including hydrogels, microneedles, nanoparticles, electrospun scaffolds and exosome-based systems, may improve drug retention in the wound, protect labile compounds from premature degradation and enable sustained, sequential or stimulus-responsive release (127–130). Such systems could potentially align drug exposure with the changing requirements of the inflammatory, proliferative and remodeling phases. In addition, local delivery may be useful for combining epigenetic modulation with antimicrobial, antioxidant, angiogenic or immunoregulatory interventions within the same wound microenvironment.

However, local delivery does not eliminate translational risks. Drug penetration may be heterogeneous in necrotic, ischemic or infected wounds, and wound exudate, protease activity, variable pH and biofilm formation may alter drug stability and release kinetics. The pharmacokinetics, tissue distribution, degradation products, sterility, batch-to-batch consistency, storage stability and tissue compatibility of the delivery materials also require rigorous evaluation (130). For exosome- and nanoparticle-based systems, additional issues include source or formulation variability, dose standardization, potency assays and scalable manufacturing. Therefore, the development of a local delivery platform should not be evaluated solely on the basis of accelerated wound closure; it should also demonstrate reproducible drug release, appropriate tissue exposure, acceptable local and systemic safety, and compatibility with routine DFU wound care.

### 4.3 Limitations of rodent models in translational interpretation

Many diabetic rodent studies evaluate relatively acute excisional wounds under controlled laboratory conditions, whereas human DFUs are chronic and heterogeneous lesions shaped by persistent hyperglycemia, peripheral neuropathy, ischemia, infection, biofilm formation, repeated mechanical stress, ageing and impaired vascular supply. In addition, rodent wounds generally close more rapidly and rely substantially on wound contraction, whereas human wound repair depends more heavily on re-epithelialization, granulation-tissue formation and coordinated remodeling (131). These anatomical, physiological and mechanical differences may influence both the activity of epigenetic regulators and the penetration, retention and efficacy of locally delivered therapeutics. Accordingly, improved wound closure or reduced inflammation in a diabetic mouse model should not be regarded as direct evidence of clinical benefit in patients with DFU. Future validation should include chronic and ischemic diabetic wound models, models incorporating infection or neuropathy where appropriate, human ex vivo wound tissues, patient-derived cells and well- characterized human DFU samples. Clinically meaningful outcomes should include durable wound closure, infection control, recurrence prevention and limb preservation rather than wound closure measurements alone.

### 4.4 Indirect lessons from clinical epigenetic therapies

Clinical studies of epigenetic therapies in oncology provide useful but indirect information regarding the feasibility, toxicity and administration of these agents. In a phase I study, systemic tazemetostat treatment demonstrated clinical activity in selected malignancies but was also associated with treatment-related adverse events and dose-limiting toxicities (126). By contrast, topical remetinostat achieved local activity in cutaneous tumors without observed systemic adverse events in an exploratory clinical study, supporting the potential value of local administration for limiting systemic exposure (132). An exploratory study of the pan-HDAC inhibitor SB939 in translocation-associated sarcomas showed disease stabilization in some patients but did not demonstrate confirmed objective responses (133). Collectively, these studies illustrate that the clinical effects of epigenetic therapeutics depend on disease context, molecular selection, dose, route of administration and treatment duration. However, they should not be interpreted as evidence that these agents are effective in DFU, because tumor biology, wound biology and the safety requirements of chronic wound treatment are substantially different.

Therefore, histone modification-targeted therapies for diabetic wounds remain at an early translational stage, progressing from mechanistic validation and proof-of-concept studies towards the demonstration of reproducible therapeutic benefit in clinically relevant models. Future development should prioritize cell- and tissue-specific target validation, stage-appropriate treatment schedules, locally controlled delivery, pharmacokinetic and biodistribution studies, and long-term safety assessment. Studies should also incorporate human-relevant models and clinically meaningful endpoints rather than relying solely on wound closure measurements. These steps will be necessary to determine whether epigenetic interventions can provide durable and safe benefits for patients with DFU.

## 5 Conclusions and future perspectives

Overall, hPTMs represent a key epigenetic regulatory layer implicated in impaired healing of diabetic ulcers. They participate in multiple processes, including the initiation and resolution of inflammation, angiogenesis, fibroblast activation, extracellular matrix remodeling and re-epithelialization. Existing studies suggest that hyperglycemia, the accumulation of advanced glycation end products (AGEs), oxidative stress and the chronic inflammatory microenvironment may collectively drive aberrant histone acetylation, methylation and other modification profiles. These alterations may, in turn, promote sustained expression of pro-inflammatory genes, dysregulated macrophage polarization, endothelial cell dysfunction and impaired myofibroblast differentiation, thereby contributing to chronic non-healing wounds. Thus, histone modifications may represent an important regulatory interface linking metabolic dysregulation to immune and tissue-repair responses, although the available evidence remains heterogeneous and predominantly preclinical.

Future studies should characterize the dynamic changes in histone modifications across cell types and healing stages in diabetic foot ulcers and identify key modifying enzymes and biomarkers associated with persistent inflammation, impaired angiogenesis and aberrant fibrosis. Local delivery systems, including hydrogels, microspheres and nanocarriers, may enhance the local activity of epigenetic therapeutics while reducing systemic toxicity. Their combination with growth factors, exosomes, cell-based therapies and anti-infective treatments also warrants further investigation.

The biomarker potential of hPTM-related signatures also warrants investigation. Histone-modification patterns measured in wound-edge or wound-bed tissue, circulating immune cells, extracellular vesicles, or cell-free chromatin may provide information about inflammatory persistence, impaired angiogenesis, infection risk, healing trajectory, or treatment response. For example, combined assessment of selected histone marks, the expression or activity of their modifying enzymes, and corresponding inflammatory or metabolic mediators may be more informative than any single hPTM measurement. However, the prognostic value of these signatures has not been established in adequately powered longitudinal DFU cohorts. Future studies should determine whether hPTM-related profiles are reproducible across wound locations, disease stages and patient populations, and whether they provide prognostic information beyond established clinical variables such as ulcer severity, perfusion, infection status and glycemic control. Standardized sampling, cell-type-resolved analysis and prospective validation will be required before hPTM-related signatures can be considered clinically useful biomarkers.
